# Determination of absorption dose in chemical mutagenesis in plants

**DOI:** 10.1371/journal.pone.0210596

**Published:** 2019-01-14

**Authors:** Changjiao Ke, Wenxiang Guan, Suhong Bu, Xiaoxu Li, Yun Deng, Zhinan Wei, Weiren Wu, Yan Zheng

**Affiliations:** 1 Key Laboratory of Genetics, Breeding and Multiple Utilization of Crops, Ministry of Education, Fujian Agriculture & Forestry University, Fuzhou, Fujian, China; 2 Fujian Provincial Key Laboratory of Crop Breeding by Design, Fujian Agriculture & Forestry University, Fuzhou, Fujian, China; 3 College of Life Sciences, Fujian Agriculture & Forestry University, Fuzhou, Fujian, China; College of Agricultural Sciences, UNITED STATES

## Abstract

Chemical mutagenesis is a useful tool for inducing mutations in plants. Seeds are often used as the material for chemical mutagenesis. The biological effect of a chemical mutagen on seeds is determined by absorption dose (the product of mutagen concentration and acting time, which starts after the mutagen is absorbed by the seeds). In practice, however, the concept of exposure dose (the product of mutagen concentration and treating time) is usually used instead because the time for absorbing mutagen is unknown. In this study, we conducted an experiment using ethyl methane sulphonate (EMS) to treat cauliflower seeds, in which five EMS concentrations (0%, 0.5%, 1.0%, 1.5% and 2.0%), three treating time lengths (4 h, 6 h and 8 h) and two pretreatments (non-presoaking and presoaking of seeds for 2 h) were set. We obtained a well-fitted nonlinear regression model for the relationship between seedling survival rate and the EMS treatment, and its marginal models for the two pretreatments. Based on the models, we determined the EMS absorption doses under the two different pretreatments and identified their 50% lethality dose (LD_50_). We found that presoaking could delay EMS absorption and therefore reduce the injury caused by EMS within a given treating time, but could hardly change the biological effect of EMS after it is absorbed. The conclusions about absorption dose and presoaking effect obtained in this study might be generally applicable to plant chemical mutagenesis in principle.

## Introduction

Mutagenesis is a powerful and effective tool for creating genetic variation, which has been widely used for genetic improvement in plants [1]. The main advantage of mutation breeding is the possibility of improving one or a few characters of a variety without changing the genetic background [2]. To achieve ideal results in mutagenesis, suitable mutagen doses are required. It is commonly considered that mutagen doses inducing 25%-50% lethality (LD_25_–LD_50_) among M_1_ plants would be appropriate because they could result in the highest mutation rates [3]. In physical mutagenesis, mutagen dose is the product of dose rate and time under constant irradiation conditions. As the biological effect of radiation appears only when the radiation energy absorbed by the organism exceeds a critical value, nowadays people usually use absorption dose (the energy absorbed per unit mass) rather than exposure dose (the dose of radiation applied) in plant mutagenesis [4].

Analogous to that in physical mutagenesis, mutagen dose in chemical mutagenesis can be defined as a product of mutagen concentration and time [5, 6]. More frequently, however, the dose of chemical mutagen only refers to the mutagen concentration under fixed treating time [1, 7–12]. Therefore, the concept of dose in chemical mutagenesis is not unified. In addition, unlike that of physical mutagenesis, the strength of chemical mutagen treatment is usually measured with exposure dose instead of absorption dose, probably because determination of the absorption dose in chemical mutagenesis is difficult. Indeed, how to determine the absorption dose of chemical mutagen remains an unsolved problem. However, it is obvious that absorption dose is also more reasonable and reliable than exposure dose in chemical mutagenesis because chemical mutagens yield biological effects only after they are absorbed by the organisms. The absorption of mutagens by organisms can be influenced by various factors such as the concentration of mutagen solution and the water content in organism tissues. The same exposure dose does not necessarily mean the same dose of mutagen received. Hence, estimation of absorption dose is required, which would help us more precisely analyze the biological effects of chemical mutagens, more reasonably compare results from different experiments, and more properly design experiments in mutation breeding programs.

In this study, we investigated the possibility of determining the absorption dose in chemical mutagenesis in plants. We carried out an experiment of treating cauliflower seeds with ethyl methane sulphonate (EMS) as an example, in which three factors including EMS concentration, treating time and pretreatment (presoaking) were tested. We established a mathematical model for the relationship of EMS effect with the three factors, from which we distinguished the absorption dose and the exposure dose of EMS mutagenesis and identified the absorption dose causing 50% lethality (LD_50_) under two different pretreatments. The results of our study put forward a method of determining absorption dose in chemical mutagenesis, which will facilitate mutation breeding in plants.

## Materials and methods

### Plant material

Cauliflower (*Brassica oleracea* L. var. *botrytis*) is an important vegetable belonging to the *B*. *Oleracea* species. Studies on chemical mutagenesis in cauliflower has been very limited so far. Hence, we chose cauliflower as the material for this study, not only taking it as a model, but also hoping to facilitate the mutation breeding of cauliflower. New harvested dry seeds of ‘White 60 Days’, a popular cauliflower cultivar in Fujian, China, were used for the experiment.

### EMS treatment and trait investigation

EMS is a common chemical mutagen used for plant mutation breeding as well as for genetic study purpose. So, we used EMS as the chemical mutagen in this study. Cauliflower seeds were either presoaked or not presoaked in distilled water at room temperature for 2 h. The seeds were then soaked in EMS solution (prepared in 0.1 M phosphate buffer, pH7.0) contained in 2 ml Eppendoff tubes (50 seeds per tube) with gentle shaking (100 rpm) at 23°C for at least 4 h. After that, the seeds were washed 3 times with distilled water and further immersed in distilled water for 2 min, followed by thorough washing with running water for 2 h. After washing, the seeds were sown immediately on two layers of wet filter paper in Petri dishes, and incubated in dark at 25°C for 7 days. Then, the dishes were placed under a photoperiod of 16 h light at 25°C and 8 h dark at 20°C for seedlings to grow further. On the 15^th^ day after sowing, the percentage of survived seedlings (survival rate, SR) was investigated.

By referring to the reported studies of EMS mutagenesis in many other crops, we set the ranges of EMS concentration and treating time to be 0–2% and 4–8 h, respectively, for this experiment. Five different EMS concentrations (0%, 0.5%, 1%, 1.5% and 2.0%) and three different EMS treating time lengths (4, 6 and 8 h) were tested. In total, there were 30 different treatments (2 pretreatments × 5 concentrations × 3 time lengths). Each treatment was replicated for three times, with 100 seeds used in each replicate. Meanwhile, an equal number of seeds soaked in distilled water for 2 h without subsequent EMS treatment were used as control.

### Statistical analysis

In each replicate, the SR of every treatment were normalized as a ratio to control. Then, the mean of three replicates of each treatment was calculated for regression analysis. The following multivariate regression model was used to fit the data:

$$y=\overset{9}{\underset{i=1}{\sum }}b_{i}x_{i}+\varepsilon$$ (1)

where y is the mean of normalized SR of three replicates of a treatment; $x_{1}\;=\;S,\;x_{2}\;=\;C,\;x_{3}\;=\;T,\;x_{4}\;=\;S^{2},\;x_{5}\;=\;C^{2},\;x_{6}\;=\;T^{2},\;x_{7}\;=\;SC,\;x_{8}\;=\;ST,\;x_{9}\;=\;CT;\;S$ is an indicator variable of pretreatment, taking 1 for non-presoaking and 2 for presoaking, respectively; C is the concentration (%) of EMS solution; T is the time length (h) of EMS treatment; $b_{i}$ is the corresponding coefficient of $x_{i}$ and ε is residual error. The model was fitted by weighted stepwise regression at a significance level of 0.05 with the reciprocal of the variance of y as the weight using the statistics software SPSS.

## Results and discussion

### Models of EMS treatment effect

The SR dramatically decreased with the increase of EMS concentration or treating time no matter the seeds were presoaked or not (S1 Table and S1 Fig). By fitting the experimental data with Eq 1, we got the following model:

$$y=1.245S-0.422S^{2}+0.066SC-0.079CT$$ (2)

The regression was a very significant (*p*-value = 1.389 × 10^−29^) with a very high coefficient of determination (*R*^2^ = 0.998), suggesting that the model fitted the experimental data very well. Hence, the model could reliably and precisely describe the relationship between SR and EMS treatment within the range of conditions set in the experiment. The three factors (*S*, *C* and *T*) were all included in the model, suggesting that they all significantly influenced the effect of EMS treatment.

Let *S* = 1 and 2 in Eq 2. We can obtain the marginal models for the non-presoaked and the presoaked seeds, respectively:

y=0.823+0.066C−0.079CT(S=1) (3)

y=0.802+0.132C−0.079CT(S=2) (4)

**Fig 1:**
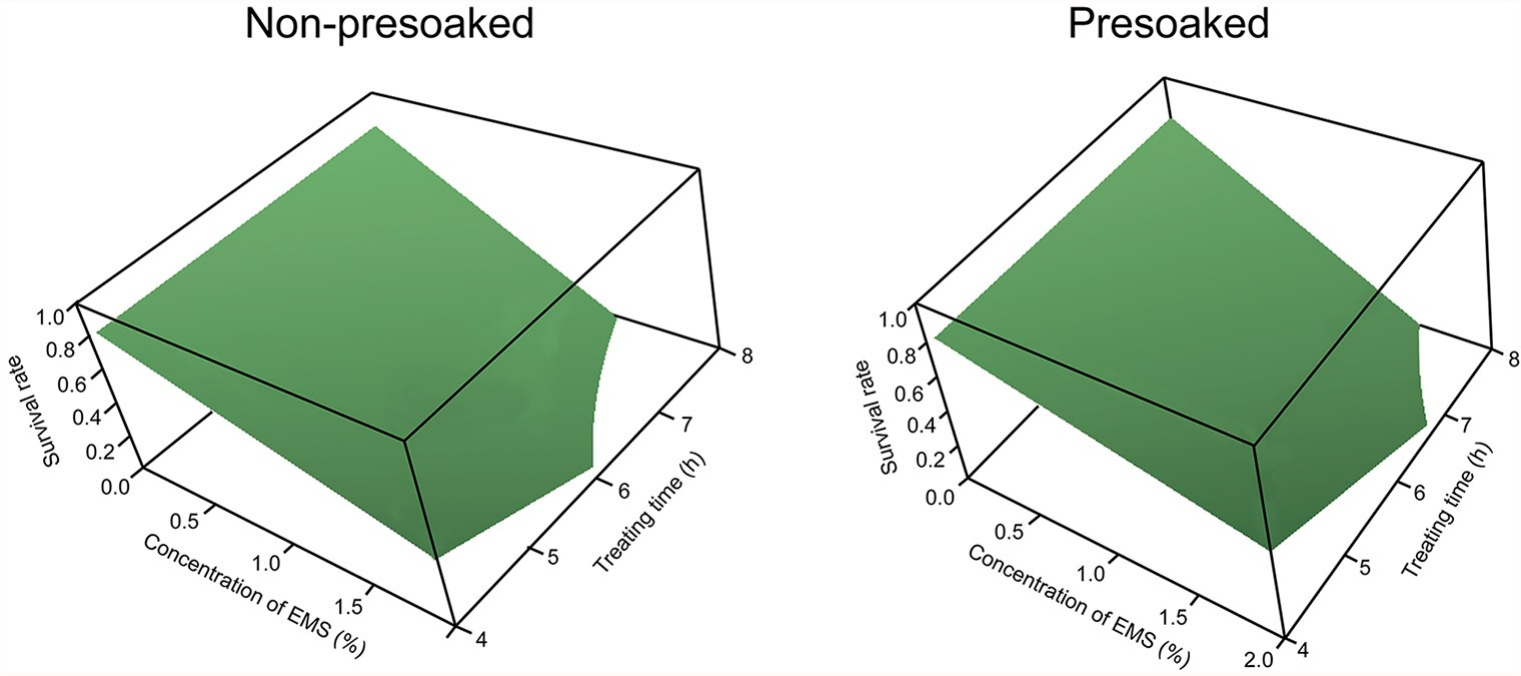
Three-dimensional surface diagrams of survival rate vs. EMS concentration and treating time. Left, non-presoaked seeds. Right, presoaked seeds.

Both Eqs 3 and 4 define an SR surface depending on EMS concentration and treating time (Fig 1). It is seen that the SR surfaces of the non-presoaked and presoaked seeds are rather similar in shape, both displaying a tendency of decreasing with the increases of EMS concentration and treating time. This suggests that EMS affects the non-presoaked seeds and the presoaked seeds in a similar way, although pretreatment may influence the effect of EMS treatment. In the rest part of this paper, we shall analyze the effect of EMS treatment based on Eqs 3 and 4.

### Dose of EMS treatment

Taking both concentration and treating time into account, the exposure dose of EMS treatment (*D*) can be defined as [5, 6]:

D=CT (5)

Substituting Eq 5 into Eqs 3 and 4, it is seen that *y* is a function of *C* and *D*. There is no one-to-one corresponding relationship between exposure dose and SR. Therefore, we cannot predict the biological effect of EMS treatment according to exposure dose.

This result is understandable. It is known that a chemical mutagen yields biological effect only after it is absorbed by the seeds [3]. Obviously, the absorption process needs a period of time (termed absorption time, *T*_a_). So, the time that the mutagen actually acts upon the seeds (termed effective time, *T*_e_) is *T*–*T*_a_. Similar to exposure dose, absorption dose (*D*_a_) can be defined as

$$D_{\text{a}}=CT_{\text{e}}=C\left(T-T_{\text{a}}\right)$$ (6)

It can be expected that the biological effect of chemical mutagen treatment will be completely determined by the absorption dose under a constant environmental condition (including temperature, pH, etc.).

Eqs 3 and 4 can be changed into the following forms:

y=0.823−0.079C(T−0.835)(S=1) (7)

y=0.802−0.079C(T−1.671)(S=2) (8)

According to Eq 6, it appears that the numbers 0.835 and 1.671 in Eqs 7 and 8 are likely to be the absorption time of the non-presoaked seeds and the presoaked seeds, respectively. Namely,

$$T_{\text{a1}}=0.835$$ (9)

$$T_{\text{a2}}=1.671$$ (10)

If this is correct, we can find the corresponding effective time

$$T_{\text{e}1}=T-0.835$$ (11)

$$T_{\text{e}2}=T-1.671$$ (12)

and the absorption doses

$$D_{\text{a}1}=CT_{\text{e}1}$$ (13)

$$D_{\text{a}2}=CT_{\text{e}2}$$ (14)

Thus, Eqs 7 and 8 can be simplified as:

$$y=0.823-0.079D_{\text{a}1}\;\;\left(S=1\right)$$ (15)

$$y=0.802-0.079D_{\text{a}2}\;\;\left(S=2\right)$$ (16)

**Fig 2:**
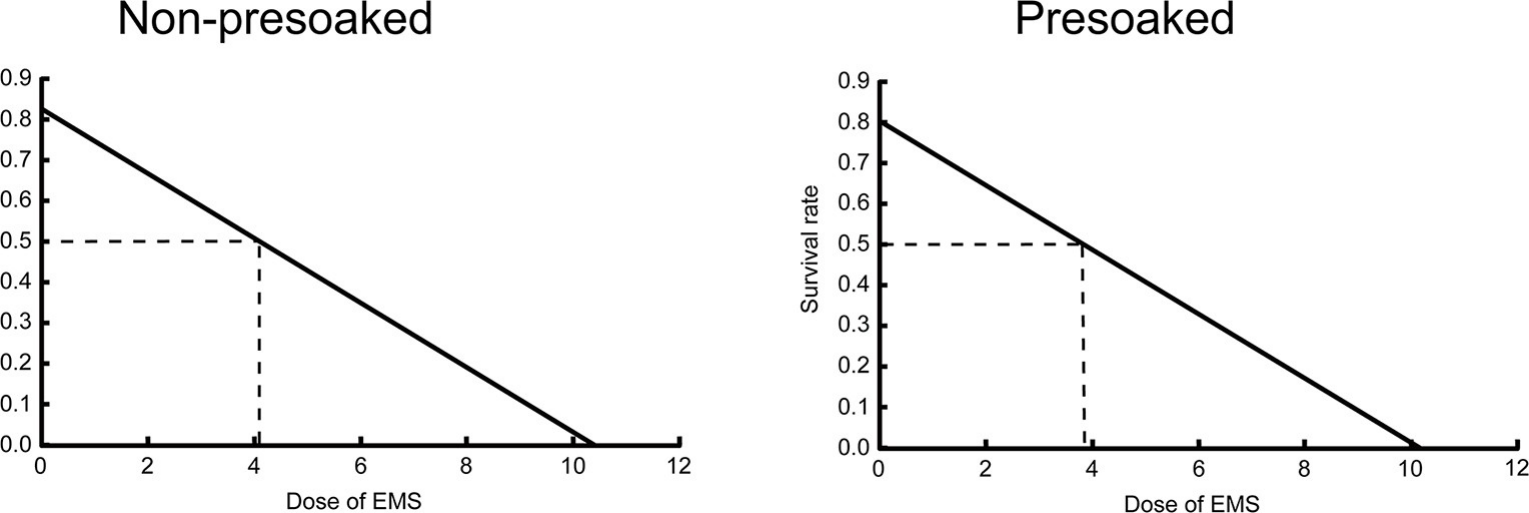
Relationship between survival rate and absorption dose. Left, non-presoaked seeds. Right, presoaked seeds. The dose causing 50% lethality is shown in each case.

Eqs 15 and 16 indicate that for both non-presoaked and presoaked seeds, SR is negatively proportional to and completely determined by absorption dose as expected (Fig 2). This suggests that the assumption of Eqs 13 and 14 is appropriate. It is noticeable that Eqs 15 and 16 are very similar in form, with very close intercepts and the same slope. This again suggests that EMS affects non-presoaked seeds and presoaked seeds in a similar way.

### Optimal concentration and time of EMS treatment

It is commonly considered that mutagen doses causing 50% reduction in seedling viability (known as LD_50_) are likely to be the most effective and efficient ones [13]. Letting *y* = 0.5 in Eqs 15 and 16, we can find that the LD_50_ of the non-presoaked seeds and the presoaked seeds are *D*_a1_ = 4.089 and *D*_a2_ = 3.823, respectively (Fig 2). Substituting the LD_50_ values of *D*_a1_ and *D*_a2_ as well as Eqs 11 and 12 into Eqs 13 and 14, we can find the constraints of *C* and *T* values for the LD_50_ of the non-presoaked and the presoaked seeds, respectively:

C(T−0.835)=4.089(S=1) (17)

C(T−1.671)=3.823(S=2) (18)

**Fig 3:**
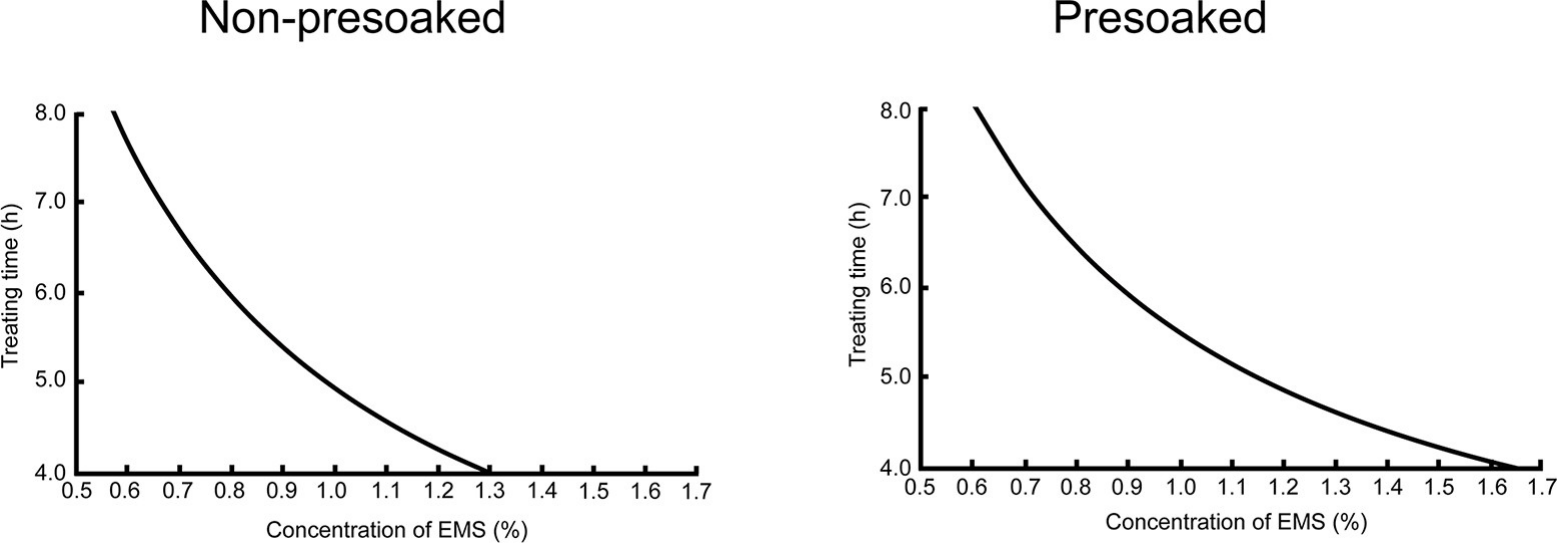
Constraint of EMS concentration-treating time combination resulting in 50% lethality. Left, non-presoaked seeds. Right, presoaked seeds.

Eqs 17 and 18 define two curves on the EMS concentration-treating time plane (Fig 3). Within the ranges of EMS concentration and treating time investigated in this experiment, any point on either of the curves determines an optimal combination of concentration and time of EMS treatment under the corresponding pretreatment (presoaking or non-presoaking). Several examples are given in Table 1.

**Table 1:** Some expected optimal EMS treatment conditions causing 50% lethality.

Treating time (h)	EMS concentration (%)	
	Non-presoaked	Presoaked
4	1.29	1.64
5	0.98	1.15
6	0.79	0.88
7	0.66	0.72
8	0.57	0.60

### Effect of pretreatment

Let Eq 3 subtract Eq 4. We can find

Δy=y(S=1)−y(S=2)=0.021−0.066C (19)

Eq 19 indicates that the difference of SR between the two pretreatments only depends on the EMS concentration, and Δ*y* < 0 in most of the cases within the range of EMS concentration investigated in the experiment (0%– 2.0%) except when *C* ≤ 0.318 (%), indicating that the non-presoaked seeds are more sensitive to EMS treatment than the presoaked seeds. This is consistent with the report that presoaking of seeds can reduce injury caused by chemical mutagens [3].

To understand the reason that the presoaked seeds are more resistant to EMS than the non-presoaked seeds, let us compare Eqs 15 and 16. As mentioned above, these two equations are very similar in form. However, it can be seen from Eqs 9–14 that *D*_a1_ is always greater than *D*_a2_ under any given *C* and *T* because *T*_a1_ is smaller than *T*_a2_. Therefore, according to Eqs 15 and 16, the SR of non-presoaked seeds should be smaller than that of presoaked seeds. So, we see that the effect of presoaking is to delay the absorption of EMS and increase the absorption time. The possible mechanism is that the water absorbed by the seeds during presoaking can impede the absorption of EMS solution and meanwhile may also dilute the concentration of EMS solution that has entered the seeds.

### Determination of absorption dose

In the definition of absorption dose (Eq 6), *C* (concentration) and *T* (treating time) are known in the experiment, but *T*_a_ (absorption time) is unknown. So, *T*_a_ is the key parameter in the determination of absorption dose. It is seen above that pretreatment can significantly affect *T*_a_. As there is only one cultivar tested in this study, it is not sure whether *T*_a_ varies among different genotypes. We expect that *T*_a_ is genetically controlled, but the genetic variation might not be very large. If this is correct, the estimates of *T*_a1_ and *T*_a2_ obtained in this study would be approximately applicable to other cultivars. Thus, for any cauliflower cultivar, the absorption dose of EMS treatment can be easily determined when the EMS concentration and treating time are given.

We have seen above that the LD_50_ of the non-presoaked seeds (4.089) and that of the presoaked seeds (3.823) are very close (Fig 2), with the former being only ~7% higher than the latter. Such a small difference is neglectable compared with the effect of EMS absorption delay due to presoaking. This suggests that while presoaking significantly affects absorption time (and therefore affects absorption dose), it hardly affects the sensitivity of seeds to absorption dose. The reason might be that the time of presoaking is not long (only 2 h), during which seeds are able to have full absorption of water but not apparently activated physiologically. This means that the EMS absorbed would have similar biological effect on the seeds no matter whether they are presoaked or not. In short, a short-time presoaking may not greatly affect the sensitivity of seeds to EMS in terms of absorption dose, although it can significantly increase the apparent resistance of seeds to EMS in terms of exposure dose. For this reason, presoaking is a useless measure for increasing mutagenesis efficiency.

Seed sensitivity to absorption dose is a genetically controlled character of a cultivar. Different cultivars can have different LD_50_. Hence, absorption dose can more accurately describe the effect of EMS and reflect the sensitivity of seeds to EMS. The theory of absorption dose established in this study should be also applicable to other chemical mutagens in principle.

## Supporting information

S1 Fig Cauliflower seedlings at the 15th day after EMS treatment. (TIF)

S1 Table Survival rate of each treatment. (DOCX)
