# Supplementary figures and images for: Determination of absorption dose in chemical mutagenesis in plants
Source: PLoS One. 2019 Jan 14;14(1):e0210596. doi: 10.1371/journal.pone.0210596 (PMC6331093; doi:10.1371/journal.pone.0210596)

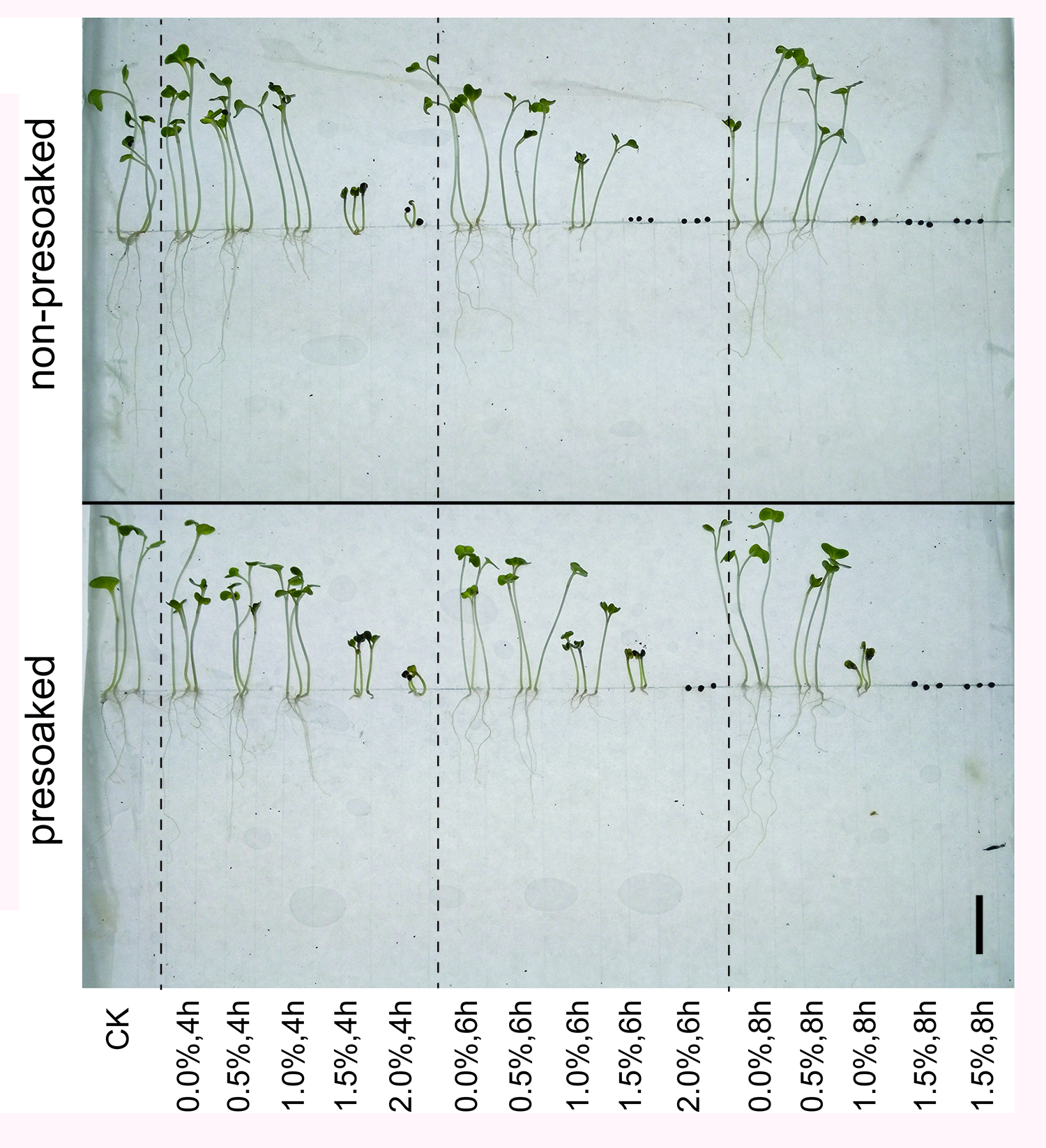

Supplement: S1 Fig — (TIF) [file pone.0210596.s001.tif]
