# Supplementary material for: Determination of absorption dose in chemical mutagenesis in plants
Source: PLoS One. 2019 Jan 14;14(1):e0210596. doi: 10.1371/journal.pone.0210596 (PMC6331093; doi:10.1371/journal.pone.0210596)
Supplement: S1 Table — (DOCX) [file pone.0210596.s002.docx]

Supplemental table S1 Survival rate of each treatment

| EMS concentration (%) | Treating time (h) | Mean ± standard deviation of survival rate (%) | |
| --- | --- | --- | --- |
|  |  | Non-presoaked | Presoaked for 2 h |
| 0 | 4 | 83.67 ± 5.19 | 85.67 ± 4.92 |
| 0.5 | 4 | 80.33 ± 5.31 | 81.33 ± 1.25 |
| 1.0 | 4 | 55.00 ± 15.75 | 57.00 ± 3.56 |
| 1.5 | 4 | 51.33 ± 11.59 | 55.67 ± 9.00 |
| 2.0 | 4 | 12.33 ± 12.82 | 18.00 ± 8.83 |
| 0 | 6 | 76.33 ± 3.09 | 81.33 ± 1.25 |
| 0.5 | 6 | 49.67 ± 8.01 | 69.33 ± 5.44 |
| 1.0 | 6 | 43.00 ± 8.60 | 52.67 ± 8.22 |
| 1.5 | 6 | 0 | 7.67 ± 7.04 |
| 2.0 | 6 | 0 | 0 |
| 0 | 8 | 76.00 ± 7.79 | 82.67 ± 6.34 |
| 0.5 | 8 | 33.67 ± 5.19 | 53.00 ± 4.32 |
| 1.0 | 8 | 1.00 ± 1.41 | 8.33 ± 7.13 |
| 1.5 | 8 | 0 | 0 |
| 2.0 | 8 | 0 | 0 |
| CK (distilled water) | 2 | 95.67±2.63 |  |
